# GTP Preference of d-Glycero-α-d-*manno*-Heptose-1-Phosphate Guanylyltransferase from *Yersinia pseudotuberculosis*

**DOI:** 10.3390/ijms21010280

**Published:** 2019-12-31

**Authors:** Suwon Kim, Mi-Sun Kim, Seri Jo, Dong Hae Shin

**Affiliations:** Graduate School of Pharmaceutical Sciences, Ewha Womans University, 52, Ewhayeodae-gil, Seoul 03760, Korea; suwon910228@naver.com (S.K.); shfwk31@ewha.ac.kr (M.-S.K.); seri9388@gmail.com (S.J.)

**Keywords:** d-glycero-α-d-*manno*-heptose-1-phosphate guanylyltransferase (HddC), *Yersinia pseudotuberculosis* (YPT), guanosine-5′-(β-amino)-diphosphate (GMPPN), guanine specificity, antibiotics

## Abstract

d-glycero-α-d-*manno*-heptose-1-phosphate guanylyltransferase (HddC) is the fourth enzyme synthesizing a building component of lipopolysaccharide (LPS) of Gram-negative bacteria. Since HddC is a potential new target to develop antibiotics, the analysis of the structural and functional relationship of the complex structure will lead to a better idea to design inhibitory compounds. X-ray crystallography and biochemical experiments to elucidate the guanine preference were performed based on the multiple sequence alignment. The crystal structure of HddC from *Yersinia pseudotuberculosis* (*YPT*) complexed with guanosine 5′-(β-amino)-diphosphate (GMPPN) has been determined at 1.55 Å resolution. Meanwhile, the mutants revealed their reduced guanine affinity, instead of acquiring noticeable pyrimidine affinity. The complex crystal structure revealed that GMPPN is docked in the catalytic site with the aid of Glu80 positioning on the conserved motif EXXPLGTGGA. In the HddC family, this motif is expected to recruit nucleotides through interacting with bases. The crystal structure shows that oxygen atoms of Glu80 forming two hydrogen bonds play a critical role in interaction with two nitrogen atoms of the guanine base of GMPPN. Interestingly, the binding of GMPPN induced the formation of an oxyanion hole-like conformation on the L(S/A/G)X(S/G) motif and consequently influenced on inducing a conformational shift of the region around Ser55.

## 1. Introduction

*Yersinia pseudotuberculosis* (*YPT*) is a Gram-negative, rod-shaped, enteropathogenic bacteria highly relevant to *Yersinia pestis* (*YP*) and *Yersinia enterocolitica* (*YE*) [1]. *YPT* causes self-limiting, ileitis, and mesenteric lymphadenitis in humans and spreads through the fecal-oral route [1,2]. Human *YPT* infection outbreaks sporadically in various parts of the world, including North America, Europe, Russia, and Japan [3]. The disease is called yersiniosis and is also triggered by *YE* [4]. Clinical symptoms of yersiniosis include abdominal pain (or occasionally ‘pseudoappendicitis’), diarrhea, and fever. Severe infection with *YPT* can lead to intestinal necrosis, hemorrhage, multi-organ abscesses, or bacteremia [2]. Around 10–30% of patients who were infected by *YPT* show the one of sequelae like reactive polyarthritis that can carry on for weeks to months [2,5].

Lipopolysaccharide (LPS) is a characteristic outer barrier of microorganisms and contributes to self-defense, membrane integrity and permeability critical for bacterial survival [6]. In particular, it constitutes the outer membrane in Gram-negative bacteria such as *Escherichia coli* (*E. coli*), *Pseudomonas*, and *Yersinia*. An outer membrane permeability barrier is vital to the antimicrobial resistance of these bacteria because they protect peptidoglycan and other intracellular targets from various antibiotics [7]. LPS is a class of large complex molecules (10–20 kDa) consisting of a highly conserved lipid A moiety for virulence on microbes, an oligopolysaccharide-rich core for connection and O-antigen units for host defense resistance [6].

The carbohydrate glycero-*manno*-heptose and its derivatives participating as building blocks of the core region are essential components of LPS in most Gram-negative bacteria. d-glycero-α-d-*manno*-heptose-1-phosphate guanylyltransferase (HddC) is the fourth enzyme involved in the heptose biosynthetic pathway and produces GDP-d-glycero-α-d-*manno*-heptose, an activated form of monosaccharides acting as glycosyl donors in glycosylation reactions. Therefore, HddC is a good target for developing antibiotics or adjuvants. Recently, the first crystal structure of the HddC family has been determined [8]. It was HddC from *YPT* (*Yp*HddC) and is the target for against yersiniosis. Its X-ray crystal structure revealed remarkable structural similarity to those of cytidylyltransferase and uridylyltransferase. Nevertheless, *Yp*HddC uses guanosine 5′-triphosphate (GTP) for catalysis rather than cytidine 5′-triphosphate (CTP) and uridine 5′-triphosphate (UTP). Despite the interesting difference, the understanding of the inclination for GTP is limited due to the absence of its crystal structure complexed with GTP. Here, we report the crystal structure of *Yp*HddC complexed with guanosine-5′-(β-amino)-diphosphate (GMPPN), a GTP analogue, to elucidate the structure-function relationship of *Yp*HddC. Based on the crystal structural data, we found a key residue to interact with GMPPN. Then, a mutation of the key residue was performed to see if the base preference would be changed.

## 2. Results

### 2.1. Macromolecule Production, Crystallization, and Structure Determination

Purified native *Yp*HddC was concentrated to 12 mg·mL^−1^. The final concentrated native *Yp*HddC and mutants seemed to be nearly 99% pure at around 26 kDa on SDS-PAGE. The initial crystallization screening was done with the native protein bound Guanosine 5′-(β, γ-imido) triphosphate (GMPPNP). Crystals of the native *Yp*HddC protein with GMPPNP appeared with long rectangular shapes in 0.05 M 4-(2-Hydroxyethyl) piperazine-1-ethanesulfonic acid (HEPES) pH 7.5, 0.2 M magnesium chloride, 1.2 M sodium citrate tribasic dehydrate. Crystallization information is summarized in Table 1.

**Figure A1:**
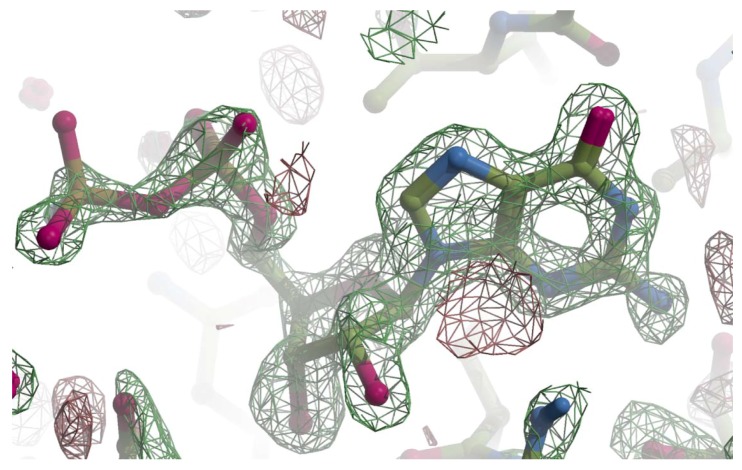
The Fo-Fc omit map for GMPPN. The Fo-Fc map was calculated with phases from the molecular replacement solution and drawn with Crystallographic Object-Oriented Toolkit (COOT) [22]. The final refined model was superimposed on the Fo-Fc map with a ball-and-stick model with the following colors; carbon is shown in green, oxygen pink, nitrogen blue, and phosphorus brown. The positive contour at 3.5 σ is represented with a green net and the negative with red.

For the X-ray data collection, various cryo-solutions were tested. However, LV CryoOil (MiTeGen, Ithaca, NY, USA) was the best cryoprotectant in this case. X-ray diffraction data were collected at a single wavelength (λ = 0.97928 Å). The crystal belongs to the primitive tetragonal space group P4_3_, with unit cell parameters a, b = 80.526 Å, c = 53.081 Å. Based on the Matthews coefficient [9], the asymmetric unit could contain one protomer with 60.5% solvent content (V_M_ = 3.11 Å^3^ Da^−1^). Details of the data-collection statistics of native *Yp*HddC with GMPPN is presented in Table 2. The phases were obtained by a molecular replacement method and refined to 1.55 Å resolution. All the residues of *Yp*HddC were visible and the model of *Yp*HddC was also well generated. The extra densities were well matched with one magnesium, one GMPPN (Figure A1), and two citrate molecules. All the residues lie in the allowed region of the Ramachandran plot and exhibited good stereochemical geometry. The structure solution and refinement data are offered in Table 3. The structural data have been deposited with the code 6JQ8 in the Protein Data Bank.

### 2.2. Overall Crystal Structure of YpHddC Complexed with Guanosine-5′-(β-amino)-diphosphate (GMPPN)

**Figure 1:**
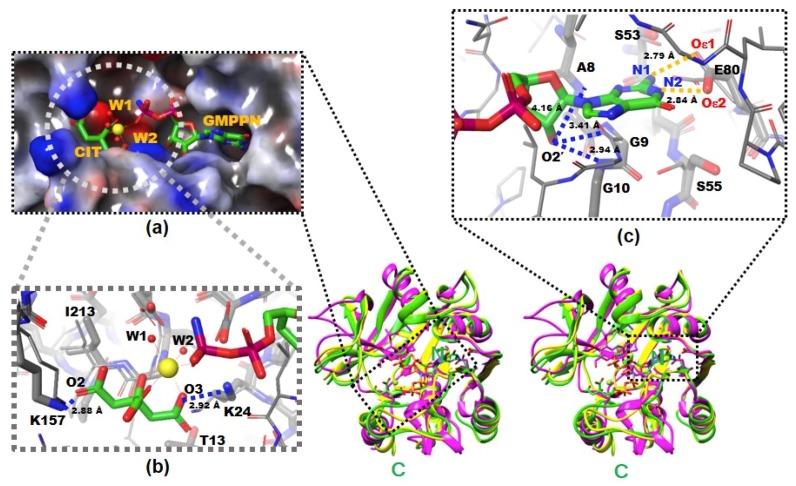
The X-ray crystal structures of native and complexed *Yp*HddC. A stereodiagram of superimposed X-ray crystal structures of *Yp*HddC complexed with guanosine 5′-(β-amino)-diphosphate (GMPPN) (**Green crystal structure**), native *Yp*HddC (**Yellow crystal structure**) and MurU (*N*-Acetylmuramic acid α-1-phosphate (Mur*N*Ac-α1-P) uridylyltransferase, **pink crystal structure**) from *Pseudomonas putida* (*Pp*MurU) has been drawn. The average RMS deviation of 218 Cα atom pairs between native *Yp*HddC and its GMPPN bound form is 0.477 Å. Those of pruned Cα atom pairs of MurU are 1.082 Å and 1.047 Å with native (123 Cα atoms) and complexed (122 Cα atoms) *Yp*HddC, respectively. The *N*- and C-terminal are labeled. (**a**) The electrostatic surface potential of the catalytic site of *Yp*HddC. The molecular surface was drawn (**Red**, **negative charge**; **Blue**, **positive charge**; **White**, **uncharged**) by the Schrödinger software package [11]. The bound GMPPN, Mg^2+^, one citrate and two water molecules are depicted with a ball-and-stick model. The six coordinates of the magnesium ion are represented by dotted lines; (**b**) the enlarged view centered on the magnesium ion (**Yellow sphere**). The native (Thin) and the GMPPN complex (Thick) structures were drawn with a ball-and-stick model. The oxygen atoms of the citrate molecule interacting with Lys24 and Lys157 are labeled. Thr13 and Ile213 interact with the citrate are also labeled. However, Leu15 is not visible in this view; (**c**) the comparison of the nucleotide-binding pockets of *Yp*HddC. The large induced-fit conformational change is observed in around the GXGXR motif of the GMPPN bound form (The **thick** model). The A8, G9, and G10 form an oxyanion hole-like conformation and interact with O2′ oxygen atom of GMPPN as indicated with the **blue** dotted lines. That change again triggers the rotation of the side chain of Ser55. The two oxygen atoms of Glu80 which form hydrogen bonds (The **gold** dot lines) with the two nitrogen atoms of the guanine base of GMPPN are also labeled.

**Figure 2:**
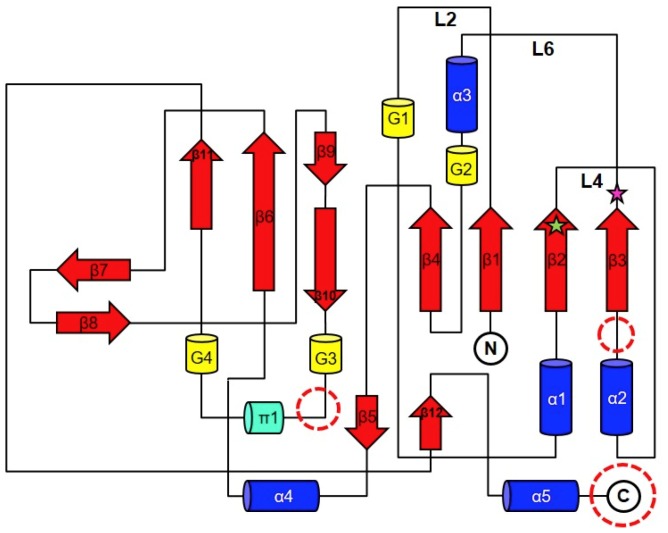
The general topology diagram of sugar nucleotidylyltransferases based on *Yp*HddC. The secondary structures were drawn and labeled with α for α-helix (**blue cylinder**), β for β-strand (**red arrows**), π for π-helix (**green cylinder**), and G for 3_10_-helix (**yellow cylinder**). The dotted circles represent where additional domains are frequently observed in the sugar nucleotidylyltransferase family. L represents a loop. The green star represents the residue, Ser53, and the pink star represents Glu80. They were the residues for the point mutation.

The crystal structure of *Yp*HddC complexed with GMPPN revealed that its overall structure is similar to that of the UDPGP family (IPR002618) classified by InterPro [10] (Figure 1). The UDPGP family catalyzing the transfer of an uridylyl group contains UTP-glucose-1-phosphate uridylyltransferases (GalU, EC: 2.7.7.9), UDP-sugar pyrophosphorylases (EC: 2.7.7.64), UDP-*N*-acetylglucosamine pyrophosphorylases (EC: 2.7.7.23) and UDP-*N*-acetylhexosamine pyrophosphorylases. In the protein family hierarchy, the UDPGP family can be classified as the subset of the homologous superfamily, nucleotide-diphospho-sugar transferases [10]. *Yp*HddC is structurally and functionally related to GalU. This family shares a unique topology of an extended β-sheet composed of nine β-strands sandwiched by several α-helices. This topology comprises a common sugar nucleotidylyltransferase (SNT) fold carrying the canonical signature motif GXGXR (^10^GLGTR^14^ in *Yp*HddC) for nucleotide-binding [8]. This core domain is extended through the addition of functional domains at several places (Figure 2). In the case of GalU from *Burkholderia vietnamiensis* (*Bv*GalU, PDB ID: 5I1F), there are two insertions: one α-helix is attached to the C-terminus (most frequently found in other members) and extra two α-helices are inserted into the loop between α2 and β3. In another case of mannose-1-phosphate geranyltransferase from *Thermus thermophilus HB8* (PDB ID: 2CU2), two α-helices are inserted at the loop between 3_10_-helix G2 and π-helix π1. It is noteworthy that there is no insertion in these places in the case of *Yp*HddC. However, *Yp*HddC shows unique local conformations in some regions. Compared with its closest homologs, it has the very simple two loops between β6 and β7 and between β8 and β9. In addition, it has the longest loop connecting β1 and α1 (Figure 2).

### 2.3. Citrates and Magnesium in the Crystal Structure of YpHddC

There were continuous electron densities from the entrance into the deep inside of the catalytic pocket of *Yp*HddC. They were well matched with citrate, magnesium, and GMPPN molecules (Figure 1a). Lys24 and Lys157 aid the binding of the citrate molecule with its oxygen atoms, O3 and O2, in the distances of 2.92 Å and 2.88 Å, respectively (Figure 1b). Thr13, Leu15, and Ile213 also participate in stabilizing the interaction of the citrate carbon backbone through hydrophobic interaction. The stabilized citrate molecule plays as a tridentate ligand and seizes a six-coordinated magnesium ion. Two neighboring water molecules and one oxygen atom from the GMPPN molecule coordinate with the magnesium ion at the opposite side of the citrate molecule. Asp210 stabilizes these two coordinating water molecules by hydrogen bonds. There is also one more citrate molecule bound in the crystal structure of *Yp*HddC. This citrate molecule is bound on the surface of the beginning region of α2 where it forms a hydrogen bond with Asp59 and interacts with Lys57 providing both charged and hydrophobic surfaces.

### 2.4. Mutation Effects on Base Specificity

**Figure 3:**
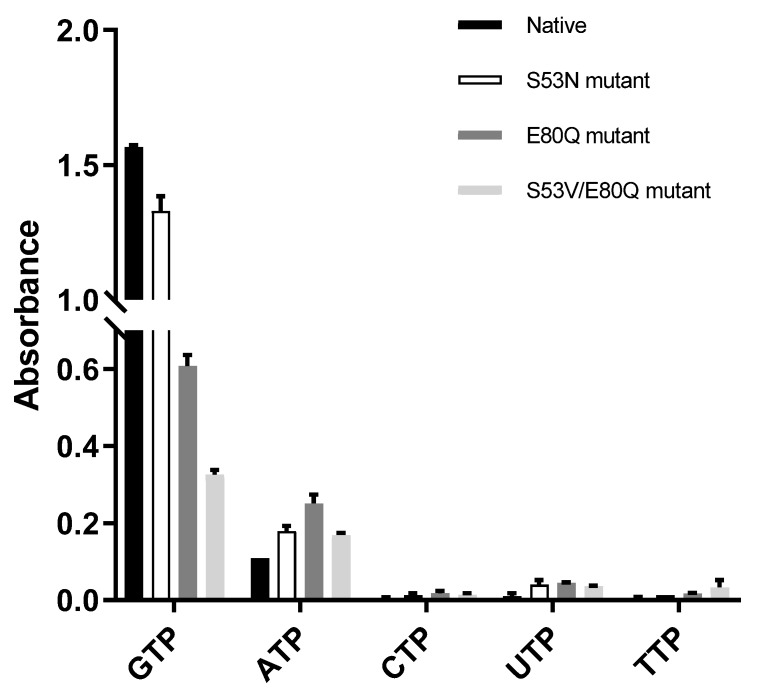
The comparison of absorbance of native *Yp*HddC, S53N, E80Q, and S53V/E80Q mutants. The malachite assay was performed with 0.25 mM NTP and 1 mM αM1P. The observed absorbance could be considered as the reactivity of HddC. Compared to native *Yp*HddC, the guanine preference of mutants fell drastically. However, pyrimidines were not recruited unlike other HddC homologs. The variation in the pyrimidine specificity of mutants seemed to be insignificant. In contrast, the usage of ATP went up a bit.

The three mutants were designed and cloned to elucidate the base preference for *Yp*HddC. An E80Q mutant of *Yp*HddC was designed to mimic uridylytransferases with a single point mutation. A double mutant (S53V/E80Q) was prepared because its active pocket is much more similar to that of the UDPGP family. An S53N mutant was also made in order to copy the active site of *Pp*MurU. With these mutants, the malachite green assay has been performed. Their activity was measured and represented as a bar graph (Figure 3). Briefly, all three mutants still showed the GTP preference (Figure 3).

## 3. Discussion

### 3.1. Effects of GMPPN in the Crystal Structure of YpHddC

There was a clear electron density that turned out to be GMPPN in the GTP binding pocket. In the protein data bank (PDB), no GMPPN molecule is searched. Therefore, in our knowledge, it is the first GMPPN structure determined by X-ray crystallography. In contrast, some of AMPPN bound structures can be searched in the PDB (PDB ID: 5KQ8, 3NSZ, 4JQE, 5J2J, 5H9B, and 3IED). The cavity is mainly composed of β1, β2, β3, and α3 together with their connecting loops L2, L4, L6 (Figure 2).

**Figure 4:**
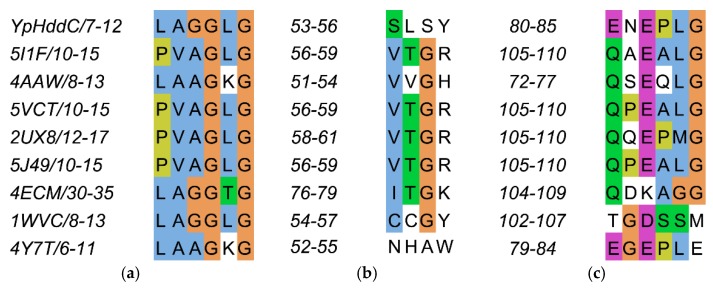
The sequence alignment of the three loops comprising the nucleotide-binding pocket of *Yp*HddC. (**a**) Loop 2 (L2); (**b**) Loop 4 (L4); and (**c**) Loop 6 (L6) are depicted in multiple sequence alignment using Jalview [12]. The top eight closest of *Yp*HddC of which the protein structures have been deposited in the Protein Data Bank (PDB) (PDB ID: 5XHW) were selected and aligned. The sequence numbers of the loops are displayed on the left side of each sequence. **Blue**, hydrophobic; **Magenta**, negative charge; **Green**, polar; **Orange**, glycines; **Yellow**, prolines; **White**, unconserved.

The residues on the loop L2 are Leu7, Ala8, Gly9, Gly10, Leu11, and Gly12 (Figure 4a). 

They are contributing to building the canonical signature motif, GXGXR. Ala8, Gly9, and Gly10 experience an induced-fit conformational change when they interact with GMPPN in the crystal structure. In detail, an oxyanion hole-like conformation was induced by the three rearranged backbone nitrogen atoms. Consequently, the induced oxyanion hole attracts the O2′ oxygen atom of the ribose moiety of GMPPN (Figure 1c). The distances between the backbone amide groups of Ala8, Gly9, Gly10 and the O2′ oxygen atom are 4.16 Å, 3.41 Å, and 2.94 Å, respectively. In contrast, this oxyanion-like architecture is not induced but already formed in the native crystal structure of MurU [13] or is absent in CMP-2-keto-3-deoxy-*manno*-octulsonic acid synthetase [14].

The residues on the loop L4 are Ser53, Leu54, Ser55 and Tyr56 (Figure 4b). In the majority of nucleotidylyltransferase, valine is the highly conserved residue at the corresponding position of Ser53 of *Yp*HddC. Interestingly, this serine residue of *Yp*HddC does not show meaningful interaction with the guanine base. Actually, the atomic positions of Ser53 and Leu54 are unaffected after the GMPPN binding (Figure 1c). Meanwhile, the hydroxyl group of Ser55 is rotated 180° to avoid the electrostatic repulsion caused by the conformational change of the backbone carbonyl group of Gly9 during the GMPPN binding. Consequently, Ser55 lies almost at the outlier of the Ramachandran plot. This kind of unusual phenomenon is not observed in other uridylyltransferases.

The residues on the loop L6 are Glu80, Asn81, Glu82, Pro83, Leu84, and Gly85 (Figure 4c). Of these, Asn81 and Glu82 locate outside the GTP binding pocket. The overall positional shift of the L6 loop during the GTP binding seemed limited. It means that the binding of the base portion of GTP follows a lock-and-key mechanism contrast to the triphosphate portion requiring an induced-fit docking. The same phenomenon is also observed in the crystal structures of α-d-glucose-1-phosphate cytidylyltransferase from *Salmonella typhi* (*St*RfbF) and *Pp*MurU of which the native and pyrimidine-bound forms have been published. The volume of each cavity for bases is around 290 Å^3^ regardless of bases when calculated with the MOLCAD module implemented in SYBYL-X 2.1.1. [15] Therefore, the base binding pockets of these enzymes are almost preformed and the sizes of cavities do not seem to have a role to distinguish between purines and pyrimidines.

L6 contains Glu80 which is a key residue to interact with GMPPN. Two oxygens of the Glu80, Oε1, and Oε2, make interactions with N2 and N1 atoms of guanine with the distances of 2.84 Å and 2.79 Å, respectively (Figure 1c). Previously, the critical role of Glu80 was predicted by multiple sequence alignment and anticipated by a manual docking of GTP with a native *Yp*HddC [8]. Glu80 is found in a unique motif EXEPLGTGGA among approximately five thousand HddC homologs. Therefore, the formation of hydrogen bonds between two oxygens of Glu80 and two nitrogen atoms of GMPPN was suggested to be a major driving force to select and interact with the guanine ring of GMPPN. Due to the binding of the citrate molecule, the spatial orientation of the pyrophosphate of GMPPN is more like that of CDP-glucose, the product form determined at the crystal structure of *St*RfbF [16].

### 3.2. Mutation Effects on Base Specificity

The GMPPN complexed structure of *Yp*HddC confirmed its GTP preference and also revealed that Glu80 is the key residue to hold GTP in the nucleotide-binding pocket. In contrast, uridylytransferases use a glutamine residue in their active site to recruit UTP as observed in *Bv*GalU. In the X-ray crystal structure of *Bv*GalU, the Nε2 and Oε1 atoms of Gln105 play a critical role in recruiting UTP. The two atoms of Gln105 make electrostatic interactions with the O4 and N3 atoms of bound UDP-glucose in the distances of 2.85 Å and 3.73 Å, respectively. Majority of the crystal structures of uridylytransferases currently deposited in the PDB confirm the presence of a glutamine residue in their nucleotide-binding site to recruit UTP.

The influence of single point mutation on the inclination of RmlA from thymidine to guanine was reported [17]. Therefore, a point mutation of Glu80 of *Yp*HddC to Gln80 has been performed to check whether a single point mutation would be enough to alter the guanine preference. Unexpectedly, the E80Q mutant augmented the inclination of adenine preference rather than uridine (Figure 3). The result could be explained, however, considering the sequence variance of the nucleotide comprising loops L2, L4, and L6 of *Yp*HddC compared with those of uridylytransferases (Figure 4). Since the result of the single point mutation did not match our expectations, a double mutant (S53V/E80Q) of *Yp*HddC was constructed to mimic the nucleotide-binding pocket of the UDPGP family. The corresponding residues of Ser53 and Glu80 of *Yp*HddC are valine and glutamine in the UDPGP family, respectively (Figure 4). The assay result, however, showed that GTP affinity was severely diminished without increment of pyrimidine affinity (Figure 3).

The last trial was done again with a single point mutant (S53N) because the top structural homolog of *Yp*HddC was *Pp*MurU [13]. The crystal structure of *Pp*MurU complexed with UTP showed that Asn52 is the key residue to hold UTP. Interestingly, Glu79 of *Pp*MurU did not interact with UTP unlike Glu80 of *Yp*HddC, where its hydrogen bonds are critical to grabbing GTP. Therefore, to mimic the UTP binding site of *Pp*MurU, the S53N mutant of *Yp*HddC was constructed and assayed. Unfortunately, this mutant also showed that GTP is still the major cofactor for the nucleotidylyltransferase activity (Figure 3). These results meant that, unlike RmlA, the GTP preference of *Yp*HddC is not determined by single but by multiple factors. The resistance against the mutations in the active pocket suggested that the active site residues of *Yp*HddC are properly designed to accommodate GTP as a crucial base.

## 4. Materials and Methods

### 4.1. YpHddC and Guanosine 5′-[β, γ-imido] Triphosphate Co-Structure Determination

#### 4.1.1. Preparation of Protein Native *Yp*HddC

The *YphddC* gene coding for the *Yp*HddC protein (NCBI Reference Sequence: WP_050090753.1) was inserted in the pBT7 plasmid DNA (pDNA) and gained from Bioneer (Daejeon, Korea). To express the protein, we transformed pDNA into *E. coli* BL21 (DE3). Transformed *E. coli* cells were incubated on Luria-Bertani (LB) agar plates. Several colonies were chosen and grown in test tubes with a cap to determine the condition for culturing in bulk. In the process, a cell stock was prepared and frozen. The mass culture proceeded at 310 K with shaking. As the absorbance at 600 nm of broth reached 0.6–0.8, Isopropyl-β-d-1-thiogalactopyranoside was put for expression of *Yp*HddC. The culture was placed back into an incubator shaker and incubated at 310 K for 3 h. To collect cells, we centrifuged the culture fluid at 7650 g (6500 rev·min^−1^) for 10 min in a high-speed refrigerated centrifuge at 277 K. The cultured cell pellet was suspended and fragmentized using a Digital Sonifier 450 (Branson Ultrasonics Co., Danbury, CT, USA). Cell debris was pelleted by centrifugation. The supernatant was affinity-purified using a HisTrap column on an ÄKTA explorer system (GE Healthcare, Piscataway, NJ, USA) based on the previous study [8]. The purified native *Yp*HddC protein containing an *N*-terminal His_6_-tag followed by 8 amino acid sequence, EFSQQDSD (MHHHHHH EFSQQDSD) was concentrated.

#### 4.1.2. Crystallization

Screening for crystallization conditions was performed with native *Yp*HddC combined with GTP analog, GMPPNP. The screening was carried out at room temperature using the sparse-matrix method [18] with several screens from Hampton Research (Laguna Niguel, CA, USA). We set up the crystallization screening with the Hanging-drop vapor-diffusion method on a VDX48 plate with sealant (Hampton Research, Aliso Viejo, CA, USA). Crystals of native *Yp*HddC combined with 2.25 mM GMPPNP and 10 mM MgCl2 were obtained in 0.05 M HEPES pH 7.5, 1.4 M sodium citrate tribasic dihydrate. To obtain high-quality co-crystals of native *Yp*HddC combined with GMPPNP, we carried out fine refinement around the condition of 0.05 M HEPES pH 7.5, 1.4 M sodium citrate tribasic dehydrate with 0.01–0.2 M magnesium chloride. Magnesium is absolutely required for catalytic activity of *Yp*HddC as a cofactor and also was essential for crystallization. Finally, co-crystals of *Yp*HddC with GMPPN, having good quality for diffraction, were obtained in 0.05 M HEPES pH 7.5, 0.2 M magnesium chloride, 1.2 M sodium citrate tribasic dehydrate.

#### 4.1.3. Data Collection and Processing

Crystals were soaked in LV CryoOil (MiTeGen, Ithaca, NY, USA) as a cryoprotectant and flash-cooled in liquid nitrogen. X-ray diffraction data were collected at a single wavelength on beamline 11 C at Pohang Light Source using a Pilatus 6 M detector (Dectris, Baden, Switzerland) placed 300 mm from the sample [19]. The oscillation range per image was 1°, with 1 s exposure. X-ray diffraction data were processed and scaled using HKL2000, iMosflm [20] and AIMLESS in the CCP4 suite [21].

#### 4.1.4. Structure Determination

Molecular replacement (MR) was performed using PHENIX [22] with the coordinates of native *Yp*HddC (PDB ID: 5XHW) as a search model. Iterative density modification, model building, and refinement were performed using the AutoBuild wizard of PHENIX. The model refinement together with the addition of water and bound molecules were performed using Crystallographic Object-Oriented Toolkit (COOT) [23] and Python-based Hierarchical ENvironment for Integrated Xtallography (PHENIX) refine.

### 4.2. YpHddC Mutation and Assay

#### 4.2.1. Plasmids and Transient Transfection

Synthesized pBT7 plasmid DNA (pDNA) containing *YphddC* gene was used as a template for constructing two single (S53N, E80Q) mutants. The sequences of the mutagenic primers of S53N mutant *YphddC* were 5′-GGGCGTGCAGCGCATTATTCTGAACCTGAGCTATAA-3′ for the forward strand and 5′-TTATAGCTCAGGTTCAGAATAATGCGCTGCACGCCC-3′ for the reverse strand. The sequences of the mutagenic primers of E80Q mutant *YphddC* were 5′-GCCCAGCGGTTCGTTCTGAATAATAAAATCCACTTTGCTGTTCACC-3′ for the forward strand and 5′-GGTGAACAGCAAAGTGGATTTTATTATTCAGAACGAAC CGCTGGGC-3′ for the reverse strand. *YphddC* mutated Glu80 to Gln80 was used as a template for constructing one double (S53V/E80Q) mutant. The sequences of the mutagenic primers of S53V/E80Q mutant *YphddC* were 5′-TCCGCTTTATAGCTCAGGACCAGAATAATGCGCTGCACG-3′ for the forward strand and 5′-CGTGCAGCGCATTATTCTGGTCCTGAGCTATAAAGCGGA-3′ for the reverse strand.

The polymerase chain reaction (PCR) and transformation were performed with the QuikChange Lightning Site-Directed Mutagenesis Kit (Agilent Technologies, La Jolla, CA, USA) as stated by the manufacturer’s protocol. The transformed *E. coli* XL10-Gold competent cells were grown in 500 µL NZ amine - yeast extract (NZY) broth and were incubated at 310 K for an hour. A 100 µL aliquot of the NZY broth was spread onto LB plates containing 50 mg·ml^−1^ ampicillin. Transformed mutant plasmids were purified using DNA-spin plasmid DNA purification kit (iNtRON Biotechnology, Seoul, Korea).

#### 4.2.2. Protein Expression and Purification for Mutants

Gene synthesis, expression, and purification of the protein was fulfilled using the same method that was published previously [8]. The expression plasmid pBT7-*YphddC* was constructed and used as a template for mutagenesis. The expression plasmids including the S53N mutant, the E80Q mutant, and the S53V/E80Q mutant, constructed using the Mutagenesis Kit, were isolated. DNA sequences were checked by gene sequencing through GenoTech corp. (Daejeon, Korea) and those recombinant plasmids were transformed into *E. coli* BL21 (DE3). The mutant proteins were purified using the same protocols used in the wild type.

#### 4.2.3. A Malachite Green Assay Method with Pyrophosphatase

A Malachite green assay method with pyrophosphatase [24] was used to study reactions with NTP (ATP, GTP, UTP, CTP, and TTP) of *Yp*HddC and its mutant. The GMP moiety from GTP is transferred to d-glycero-α-d-*manno*-heptose-1-phosphate by *Yp*HddC. Consequently, GDP-d-glycero-α-d-*manno*-heptose and pyrophosphate (PPi) are released. Instead of d-glycero-α-d-*manno*-heptose-1-phosphate, we used α-d-Mannose-1-phosphate (αM1P) purchased from Sigma for a substrate. PPi was degraded by inorganic pyrophosphatase (IPP) and detected using the malachite green reagent [25]. A detection reagent was prepared by mixing 4.2% (*w*/*v*) ammonium molybdate ((NH_4_)_6_Mo_7_O_24_) in 4 N hydrochloride, 1% (*v*/*v*) Tween 20 and 0.045% (*w*/*v*) malachite green solution in the ratio 1:3:0.1 and stirring for 30 min at room temperature (RT). The detection reagent was filtered with a 0.2 μm PVDF syringe filter (Younginfrontier Inc., Seoul, Korea) and stood for 1 h at RT before use. For NTP reaction study, reaction solutions were containing 50 mM Tris-HCl (pH 7.5), 10 mM MgCl_2_, 0.04 unit IPP and HddC (0.05 mg·ml^−1^). In addition, substrates of *Yp*HddC and its mutants, NTP (0.25 mM) and αM1P (1 mM), were used. After incubation at 25 °C for an hour, 40 μL of reaction mixtures were mixed with 160 μL of the color reagent. The mixtures were waited for 15 min to react. The absorbance of the final mixtures was measured at the specific wavelength (660 nm) using a microplate spectrophotometer (Spectramax 190, Molecular Devices Corporation, Sunnyvale, CA, USA). The comparative bar graph was worked with the GraphPad Prism 7.03 (GraphPad Software, San Diego, CA, USA).

## 5. Conclusions

In summary, the first X-ray crystal structure of GMPPN bound from among the HddC family was determined. The main molecular property of *Yp*HddC is its GTP preference regardless of overall structural similarity to the UDPGP family. Glu80 of *Yp*HddC is the key residue for the GTP affinity as revealed by E80Q and S53V/E80Q mutants. Other minor conformational changes seem to be important for nucleotidylyltransferase activity. The continuous intuitive mutant experiments based on the multiple sequence alignment together with the active site structural comparison were carried out to endow pyrimidine affinity for *Yp*HddC. Instead of discovering key residues for pyrimidine affinity, however, the results could confirm the importance of Ser53 and Glu80 of *Yp*HddC for its GTP preference. All mutants indicated that key residues holding different bases in nucleotidylyltransferase seem to be very unique and thus their base preference could not be easily copied. A further mutational and functional study is going on to alter the GTP affinity of *Yp*HddC.

## Figures and Tables

**Table 1:** Co-crystallization of *Yp*HddC and Guanosine 5′-(β, γ-imido) triphosphate (GMPPNP).

	Conditions
**Method**	Hanging drop vapor diffusion method
**Plate type**	VDX48 plate (Hampton Research)
**Temperature (K)**	296
**Protein concentration (mg·mL^−1^)**	12
**Buffer composition of protein solution**	20 mM Bis-Tris pH 7.0, 10 mM MgCl_2_, 2.25 mM GMPPNP.
**Composition of reservoir solution**	0.05 M HEPES* pH 7.5, 0.2 M magnesium chloride, 1.2 M sodium citrate tribasic dehydrate
**Volume and ratio of drop**	1.6 μL, 1:1
**Volume of reservoir**	200 μL

* HEPES: 4-(2-Hydroxyethyl) piperazine-1-ethanesulfonic acid.

**Table 2:** Data collection and processing.

	Data
**Diffraction source**	PAL/PLS beamline 11C
**Wavelength (Å)**	0.97928
**Temperature (K)**	100
**Detector**	PILATUS3 6M
**Crystal-detector distance (mm)**	300
**Rotation range per image (°)**	1
**Total rotation range (°)**	360
**Exposure time per image (s)**	1
**Space group**	P4_3_
**a, b, c (Å)**	80.526, 80.526, 53.081
**Resolution range (Å)**	50.00–1.546 (1.601–1.546)
**Total No. of reflections**	96,119 (4037)
**No. of unique reflections**	49,164 (4505)
**Completeness (%)**	98.7 (85.4)
**Redundancy**	12.4 (9.4)
**〈** **I/σ(I)** **〉**	43.89 (2.85)
**R_merge_ (%)**	5.6 (50.3)

**Table 3:** Structure solution and refinement.

	Data
**Resolution range (Å)**	44.32–1.546 (1.601–1.546)
**Completeness (%)**	96.19 (85.47)
**σ cutoff**	0 σ(F)
**No. of reflections, working set**	47,959 (4242)
**No. of reflections, test set**	1941 (171)
**Final R_work_**	0.2065 (0.2332)
**Final R_free_**	0.2276 (0.2558)
**No. of non-H atoms**	
**Protein**	1785
**Ligand**	55
**Water**	156
**Total**	1996
**RMS deviations**	
**Bonds (Å)**	0.008
**Angles (°)**	1.27
**Average B factors (Å2)**	
**Protein**	20.71
**Ligand**	21.62
**Water**	25.77
**Ramachandran plot**	
**Most favoured (%)**	97.76
**Allowed (%)**	2.24

Values for the outer shell are given in parentheses.

## Abbreviations

COOT	Crystallographic Object-Oriented Toolkit
CTP	Cytidine 5′-triphosphate
GTP	Guanosine 5′-triphosphate
GMPPN	Guanosine-5’-(β-amino)-diphosphate
HddC	d-glycero-α-d-*manno*-heptose-1-phosphate guanylyltransferase
HEPES	4-(2-Hydroxyethyl) piperazine-1-ethanesulfonic acid
LPS	Lipopolysaccharide
NZY	NZ amine - yeast extract
PHENIX	Python-based Hierarchical ENvironment for Integrated Xtallography
UDPGP	UTP-glucose-1-phosphate uridylyltransferases/UDP-glucose pyrophosphorylase
UTP	Uridine 5′-triphosphate
YPT	*Yersinia pseudotuberculosis*
YP	*Yersinia pestis*
YE	*Yersinia enterocolitica*
